# Drug-related celebrity deaths: A cross-sectional study

**DOI:** 10.1186/s13011-016-0084-z

**Published:** 2016-12-09

**Authors:** Johannes M. Just, Markus Bleckwenn, Rieke Schnakenberg, Philipp Skatulla, Klaus Weckbecker

**Affiliations:** Bonn University Clinic, Institute of General Practice and Family Medicine, Sigmund-Freud-Street 25, 53127 Bonn, Germany

**Keywords:** Addiction, Celebrity, Opioids

## Abstract

**Background:**

Celebrities are at risk for premature mortality as well as drug-related death. Despite being a vulnerable patient group, celebrities influence people’s health behaviours through biological, psychological and social processes. Therefore, celebrity endorsement of the topic could be one way to challenge the current “opioid endemic”. Our aim was to better understand the factors surrounding drug-related celebrity deaths by investigating the incidence as well as substances used between 1970 and 2015 using a cross-sectional study design.

**Method:**

We searched public databases for drug-related celebrity deaths between 1970 and 2015. They were categorized for sex, profession, age at death, year of death and substances involved. The main outcome measures are descriptive values including number of drug deaths per year and substances involved. Secondary outcome measures are analytical questions to examine whether and which factors influence age at death and year of death (e.g. type of substance use disorder).

**Results:**

We identified 220 celebrities who died a drug-related death with a clear indication of involved substances between 1970 and 2015. The average age at death was 38.6 years; 75% were male. Most celebrities died between the age of 25 and 40. The number of drug-related deaths increased in the 21st century, with a significant increase in the use of prescription opioids. Deaths involving prescription opioids and heroin were associated with a significantly lower mean age at death compared to deaths where these substances were not involved.

**Conclusions:**

Compared to the 20th century, the total number of celebrities who died from a drug-related death in the 21st century increased, possibly due to an increased involvement of prescription opioids. Negative effects on individual health decisions of celebrity’s followers could be the result.

## Background

Celebrities are at risk for premature mortality as well as drug-related death [1]. Drug-related deaths occur regularly and attract significant media coverage [2]. It was shown, that rock/pop star mortality increases relative to the general population with time since fame [3]. In professional musical performance artists, performance-related musculoskeletal pain is a common disorder and is associated with depression and music performance anxiety [4]. Actors also show an increased prevalence of musculoskeletal pain [5]. In professional athletes substance use rates are higher than in non-athletes [6].

Reasons for starting “drugs of abuse” involve the performance enhancer model (enhance strength and endurance, fight pain, reduce weight) and the abuse and addiction model (reduce stress, boost confidence, relieve craving) [6]. Many of these motives apply to celebrities as they are under constant pressure to perform and to appear healthy and productive. Opioids have a soothing effect on many of these issues. Therefore the risk for developing an addiction may be generally elevated for celebrities, especially in those with an increased prevalence of musculoskeletal pain [4–8].

Many beloved actors and musical performance artists have succumbed to an addiction involving both prescription and illicit drugs. It has been argued that in the past overdoses taken by celebrities almost inevitably involved illicit drugs such as heroin or cocaine (Janis Joplin, John Belushi, River Phoenix and others) while recently more and more celebrities have died of prescription drug overdose [2].

One of the most recent deaths involves the famous musician Prince who accidentally overdosed on fentanyl [9]. According to news media reports, attempts to start him on addiction treatment were about to commence when the tragic overdose death occurred [10].

This perceived development is parallel to trends in the general public. The incidence of deaths caused by prescription drugs in the US has increased dramatically in the 21st century. Since the year 2000, the rate of drug overdose deaths involving prescription drugs and prescription opioids has approximately tripled in the U.S. [11].

There are clear and deeply rooted biological, psychological and social processes that explain how celebrities influence people’s health behaviours [12].

Celebrity’s medical advice as well as stories about their health issues and deaths diffuse through social networks. Celebrity endorsement can act as a signal of credibility and may catalyse herd behaviour. People are classically conditioned to react positively to celebrity’s advice and desirable celebrity attributes get assigned to products and ideas [12].

Therefore, by promoting their lifestyles and beauty ideals amongst other factors, they probably exact an impact on health-related life choices [12]. As an extreme example, media reports on celebrity suicide are associated with increases in suicides in the general population in North America, Asia and Europe [13]. This was dubbed the Werther effect, following Goethe’s novel The Sorrows of Young Werther.

The aim of this study was to systematically analyze drug-related celebrity deaths to learn more about this special population as well as the substances responsible for the deaths. We hypothesized, that the number of drug-related celebrity deaths as well as the involvement of prescription opioids has increased over time.

## Method

We performed a retrospective cohort study using publicly available online information in June 2016.

We searched the Internet via the Google search engine with the term “drug-related celebrity deaths”, which yielded approximately 6 700 000 results. The most comprehensive list of drug-related celebrity deaths was found on Wikipedia and contained 451 entries [14]. When comparing to the two second largest lists (ranked.com and drugs.com, both containing 200+ entries) we found only one additional entry that met our inclusion criteria. We therefore concluded that the Wikipedia list contained most relevant entries.

All celebrities that died of an overdose between 1970 and 2015 were included. The death had to be caused by a drug itself or a combination of drugs. We defined “celebrity” as a person that (i) had a Wikipedia entry and (ii) had a Wikipedia entry devoted to her or him due to their own, substantial achievements. All cases without references, as well as those in which the substance or circumstance of death was unclear, were excluded.

The individual cases were categorized for: year of death, age at death, sex, profession, type of drug used and nationality.

In order to minimize bias, we chose a time frame (1970–2015) for which many witnesses are still alive and can reasonably contribute to public databases from their own recollection. Also, all references mentioned on the Wikipedia site were verified and compared to other available online information on the individual celebrity death.

We then performed statistical analysis using IBM SPSS Statistics 22®. After data validation and plausibility checks, descriptive statistics were conducted. In addition, Student’s *t*-test for independent samples was used to compare mean age at death as well as year of death of users/non users of the different specified drugs. Because of multiple comparisons, significance was set at *p* < 0.01 using the Bonferroni correction.

Ethical approval was not required as all data were accessed through publicly available materials and no (living) human subjects were involved.

## Results

We found 295 celebrities who died of a drug overdose between 1970 and 2015. Of these, 220 met our inclusion criteria. The sample characteristics are displayed in Table 1. In many cases, celebrities had to be excluded because the prescription drug used was not specified and couldn’t be verified elsewhere (35 out of 75 = 46.7%). In these cases, vague terms like “prescription pain killer” or “tranquilizer” were used.

**Table 1:** Sample Characteristics

Number of subjects (*n*)	220
Sex (percentage of male subjects)	75
Age at death in years (mean, SD)	38.6 (12,1)
Year of death (mean, SD)	1995 (13)
Nationality (percentage of US citizens)	65.5

**Fig. 1:**
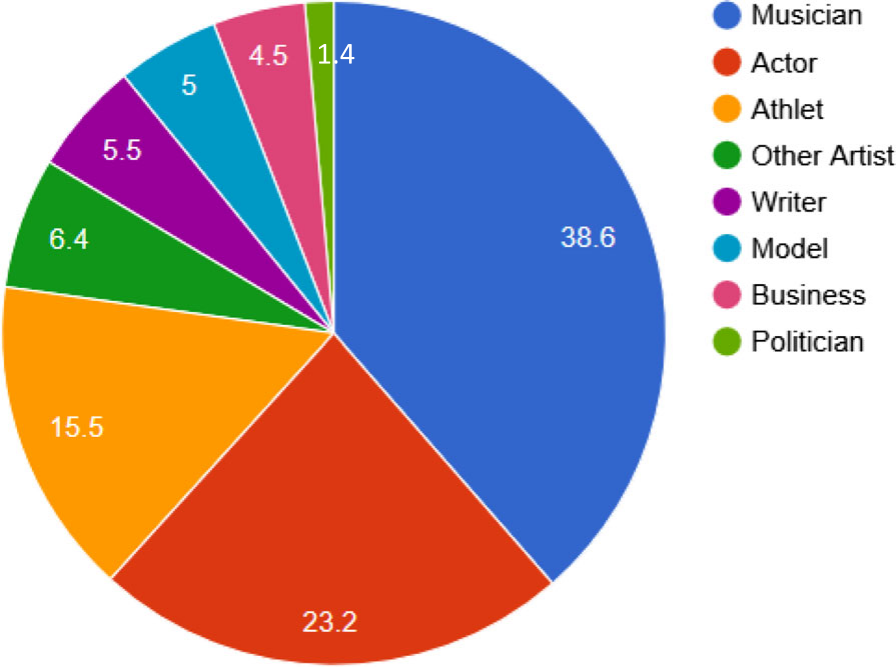
Professions of celebrities who died in relation to drug abuse in per cent between 1970 and 2015

More than half of the celebrities included were from the entertainment industry (Fig. 1). Of all celebrities categorized as actors, 13.7% worked in the pornographic film industry. In the case of athletes, professional wrestlers, rugby players, American football players and jockeys appeared more frequently on the list than others.

**Fig. 2:**
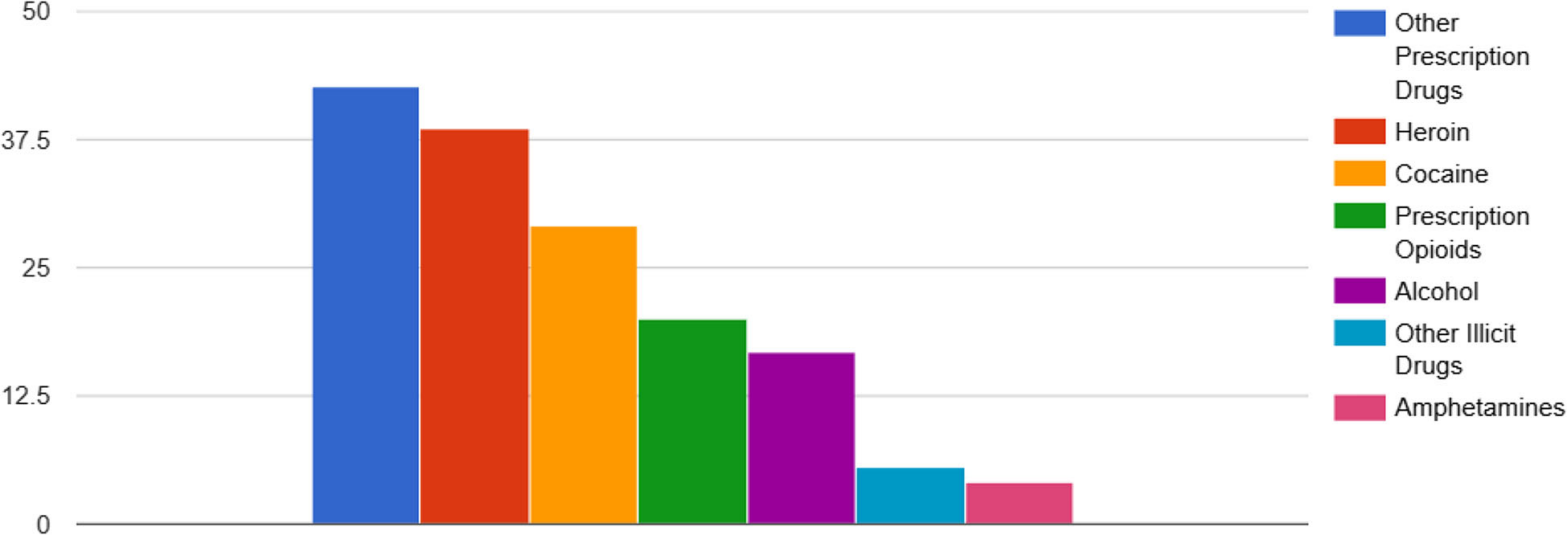
Substances used by celebrities who died in relation to drug abuse in per cent between 1970 and 2015

A percentage of each substance group associated with celebrity deaths is given in Fig. 2.

**Fig. 3:**
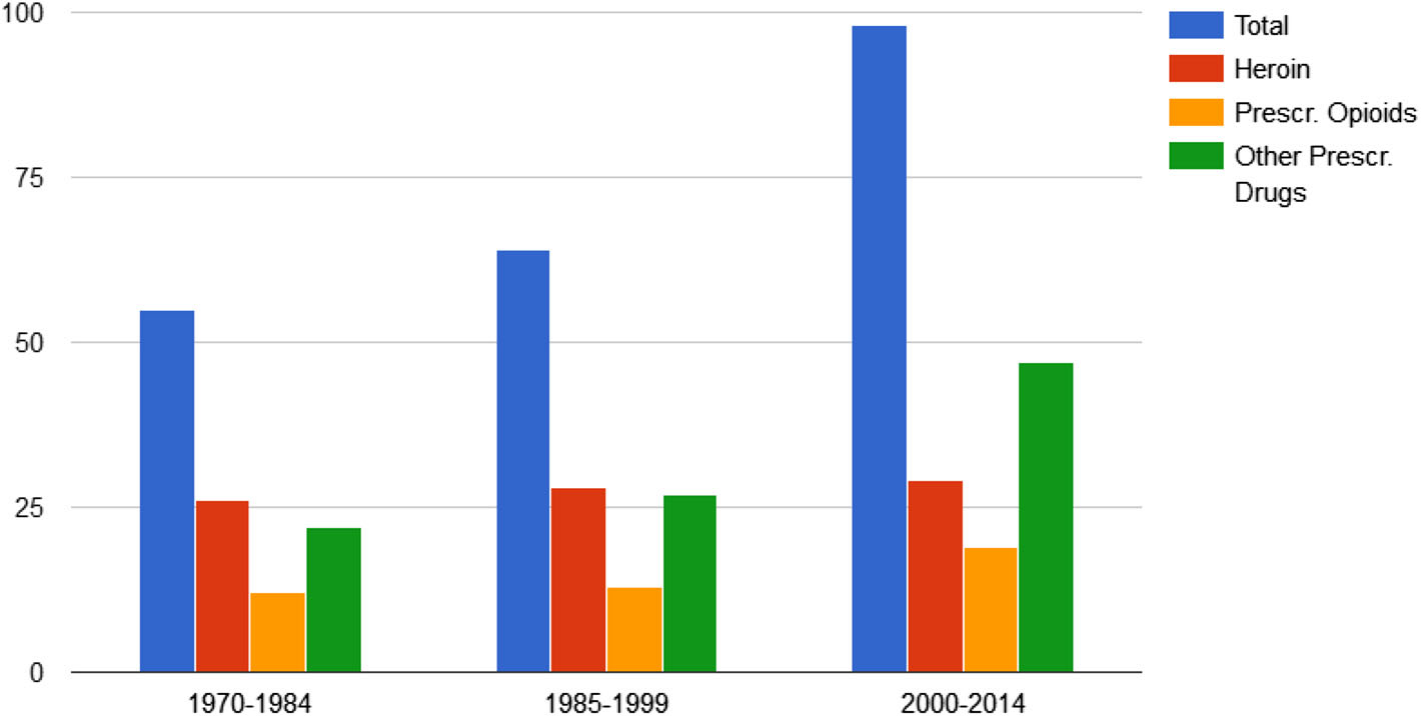
Total number of deaths per time frame and substances involved between 1970 and 2014

The number of drug-related celebrity deaths has nearly doubled within the 21st century. While the number of heroin-related deaths remained relatively stable, the number of deaths in which prescription drugs, alcohol and prescription opioids played a role has been increasing since the year 2000 (Fig. 3).

When comparing involvement and non-involvement off different drugs in celebrity deaths, we found significant differences regarding mean age at death and year of death. Prescription opioids and heroin were associated with a lower mean age at death, an opposed connection was found for other prescription drugs (Table 2). The mean year of death was more recent when prescription opioids were involved, an opposed connection was found for cocaine and other prescription drugs (Table 2).

**Table 2:** Differences regarding age at death / year of death depending on the drug involved

	Mean age at death			Mean year of death		
	Mean (SD)	CI 95% of mean difference	Significance of *t*-test ^a^: *p*-value	Mean (SD)	CI 95% of mean difference	Significance of *t*-test^a^ *p*-value
All (ages 18–82)	38.6 (12.1)	n/A	n/A	1995 (13.0)	n/A	n/A
Heroin use (yes/no)	34.8 (8.9)	−8.9–−3.1	*p* < 0.01	1995 (12.3)	−3.9–3.3	*p* = 0.868
Prescription opioid use (yes/no)	35.4 (7.7)	−7.0–−1.0	*p* = 0.01	2003 (7.9)	7.7–13.9	*p* < 0.01
Other prescription drug use (yes/no)	41.9 (13.1)	2.4–9.0	*p* < 0.01	1993 (14.2)	−3.3–0.9	*p* = 0.13
Cocaine use (yes/no)	37.3 (10.9)	−5.4–1.7	*p* = 0.297	1991 (10.3)	2.3–8.9	*p* < 0.01

^a^ *T*-test for independent samples was conducted to examine the mean difference of age at death and mean year of death in users and non-users

Sample size: 220; Degrees of freedom (df) 218

**Fig. 4:**
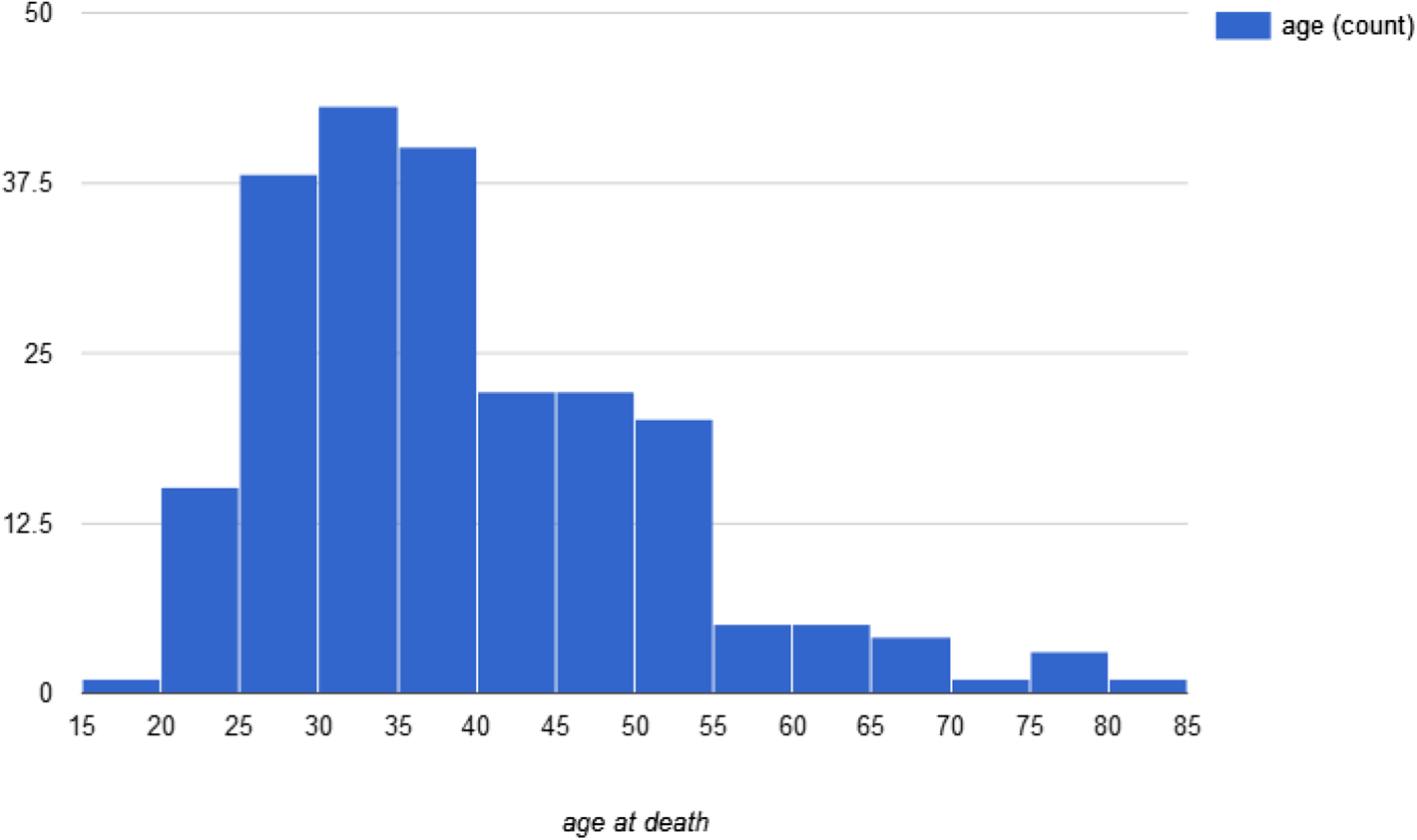
Histogram of age at death of celebrities who died in relation to drug abuse from 1970 to 2015

Most drug-related celebrity deaths occurred in the age range of 25 to 40 years (Fig. 4).

## Discussion

Our results showed an increase of drug-related celebrity deaths in the 21st century with a statistically significant increase of prescription opioid involvement. Also we were able to show that prescription opioids as well as heroin were significantly associated with a younger age at death compared to all drug-related celebrity suicides, while other prescription drugs were significantly connected with an older age at death.

There are several possible limitations to this study. We searched a U.S.-based search engine and websites in English which may have led to a selection bias towards U.S. celebrities. Also the perception of who we regard as celebrity might have changed during the study period, even more so after the rise of social media. Reporting bias might be present as historic reporting of cause of death may have changed over the study period. Pain medications and suicides may have been underreported in early years due to social acceptability issues, resulting in an apparent increase in those deaths in later years. This effect could have been reinforced by the improvement of toxicological identification methods during the study period. This might have significantly changed the results. Also we cannot control for the total number of celebrities which is most likely on a continuous rise as the entertainment industry itself is continuously growing [15]. This is why we cannot claim that the percentage of celebrities, who suffered a drug-related death, has been rising.

The increased use of prescription opioids in the U.S. in the 21st century corresponds to our finding that the involvement of prescription opioids was significantly associated with a more recent year of death.

The reasons for this increase in prescription opioid related deaths are manifold and involve high prescription rates. From the 1990s, efforts to improve pain therapy did not adequately take into account opioids’ addictiveness and the lack of documented effectiveness in the treatment of chronic pain [16]. Also, aggressive and sometimes misleading marketing of long-acting opioids to physicians increased prescription rates. We made a distinction between prescription and illicit opioids, although this distinction will be increasingly hard to maintain. Between 2013 and 2014 drug overdose deaths in the general public involving synthetic opioids increased even more rapidly, most likely due to “illicit fentanyl” entering the black market. The term “illicit fentanyl” refers to a non-pharmaceutical version of prescription fentanyl and is manufactured in illegal laboratories. Toxicology tests used by coroners and medical examiners are unable to distinguish between prescription and illicit fentanyl. It is often combined with heroin or sold as heroin, posing a lethal threat to the inexperienced user [17].

Of the 220 deaths examined, 75% occurred in men. Also, more than half of the celebrities involved were actors or musical performance artists. Athletes involved in sports with a high injury hazard (i.e. wrestling, American football, rugby) seem to be a vulnerable group [18]. Furthermore, with 13.7% of the deceased actors in our study found to be in the pornographic film industry, our data support the findings of earlier authors who found an increased likelihood for drug abuse in male and female porn actors [19, 20].

Age-based, the highest number of drug-related deaths was found in the groups aged 25 to 40. This differs from drug-related death rates in the US general population, where rates were highest among persons aged 45–54 years in 2014 [21].

Heroin was labelled the most harmful of all drugs by an expert panel considering the physical harm caused to the individual user, the tendency of the drug to induce dependence as well as the effect of drug use on families, communities, and society [22]. Our data showed that both heroin and prescription opioids were associated with a younger age at death compared to those drug-related deaths where no heroin or opioids were found. This finding does stress the importance of prevention, diagnosis and treatment of opioid addiction [23].

Our analysis has direct implications for the care of a very small but influential patient group as celebrities most likely influence the public’s health decisions. A meta-analysis showed that following an entertainment celebrity’s suicide, suicide rates in the public increased significantly in North America, Asia and Europe [13]. Consequently, it is reasonable to believe, that successfully treating a celebrity’s addiction might have a positive effect on the drug related behaviour of her or his followers. Celebrity endorsement successfully changed health-related behaviour in other contexts. When journalist Katie Couric televised her colonoscopy on NBC’s Today Show in 2000, screenings for colorectal cancer by 400 American endoscopists increased by 21% the next month [24]. While there is no similar evidence concerning drug-related behaviour, imagine the positive effects of Prince receiving a timely and adequate addiction therapy, overcoming his addiction and talking openly about his experiences.

## Conclusion

Celebrities deserve excellent healthcare, for their own good as well as for the sake of their followers. Physicians that care for celebrities should consider that celebrities may be at increased risk for substance abuse and addiction.

In the case of prescription opioids, the “Center of Diseases Control” guidelines should be complied with. If addiction is present, adequate treatment should be suggested. Facing pain, depression, stage fright and other existential issues is a challenge to many performing in the arts [4]. Doctors should be aware that neither intense suffering nor their intention to help does justify the use of prescription opioids in the absence of a clear medical indication. Thus they can prevent addiction in their celebrity patients and minimize negative public health effects for millions of their followers.
